# Secondary thoracolumbar deformity and sagittal imbalance due to osteoporosis in a young man with Cushing’s disease: A case report

**DOI:** 10.1016/j.ijscr.2020.09.182

**Published:** 2020-09-29

**Authors:** José Ramírez-Villaescusa, David Ruiz-Picazo, Cristina Lamas Oliveira, Carlos Morillas-Ariño

**Affiliations:** a Complejo Hospitalario Universitario de Albacete, Department of Orthopedics, Spine Unit, Albacete, Spain; b Complejo Hospitalario Universitario de Albacete, Endocrinologist Department, Albacete, Spain; c Hospital Universitario Dr. Peset, Department of Orthopedics, Chief of Endocrinologic Department, Valencia, Spain

**Keywords:** Cushing’s disease, Multiple vertebral fractures, Sagittal imbalance, Surgical treatment

## Abstract

**Introduction:**

To describe an unusual case of Cushing’s disease with spontaneous axial pain due to multiple consecutive vertebral fractures which led to secondary deformity that required surgical treatment.

**Presentation of case:**

A 43-year-old man was referred to our service with back pain without previous trauma. He was diagnosed of refractory arterial hypertension and we observed centripetal obesity during exploration. With clinical findings and laboratory studies, ACTH-dependent Cushing’s syndrome due to a pituitary microadenoma was diagnosed and the patient underwent an endoscopic-assisted endonasal transsphenoidal resection. Dual energy X-ray absorptiometry (DXA) revealed spine and hip osteoporosis. Moreover, X-ray, MR and CT showed multiple vertebral osteoporotic compression fractures in thoracic and thoracolumbar area. Secondary kyphosis thoracolumbar deformity and sagittal imbalance was treated by two-level Smith-Petersen osteotomies (SPO) and instrumented posterolateral arthrodesis T10-L3 using fenestrated pedicles screws with polymethyl methacrylate (PMMA). At six years of follow-up dual energy X-ray absorptiometry (DXA) recovered normal values (T-score lumbar spine L2–L4 1.4 and T-score hip -1.9) and X-ray study showed an adequate sagittal vertebral axis.

**Discussion:**

Osteoporosis is a common feature of CD and fractures occur in 30–50% of cases. Treating the underlying cause reduces the risk of new fractures. Medical therapy is usually enough but consecutive multiple vertebral fractures related to glucocorticoid excess may lead to secondary painful deformity.

**Conclusion:**

Vertebral compression fractures result from secondary corticoid-induced osteoporosis in Cushing’s disease. Early detection and treatment of primary disease decreases the risk of new fractures. However, unusual secondary spinal deformity or disability may require surgery.

## Highlights

• Vertebral fractures are frequent due to osteoporosis in Cushing disease.
• Treating de primary cause of the disease reduces the risk of new fractures.
• Surgical treatment may be needed for secondary deformity.

## 1 Introduction

Endogenous Cushing’s syndrome (CS) is a rare disease. In a European population-based study, the annual incidence was 2–4 cases per million inhabitants per year [1]. Cushing’s disease (CD) due to ACTH pituitary hyperproduction is the most frequent cause of endogenous CS and 1.2–1.7 cases per million per year have been reported, about three times more frequent than the one caused by an adrenal tumour [1,2]. CS due to an adrenal or pituitary tumour occurs mainly between 25 to 45-years, with the female-to-male incidence ratio being approximately 5:1.

In CD there is a pituitary ACTH hypersecretion and increased ACTH plasma levels, originated by a pituitary adenoma, often undetected through image techniques, which stimulate bilateral adrenal hyperplasia with cortisol hypersecretion and consequently cortisol circadian rhythm lost [3]. CD signs and symptoms depend directly on persistently high cortisol plasma levels, most of them being present in general population (arterial hypertension, central obesity, diabetes or insulin resistance) so it is often difficult to make a diagnosis [3]. Early diagnosis is important as the high rate of comorbidity and mortality in CD patients can be avoided if treated at an early stage.

Bone disease a common feature of CS and there is a variable risk of osteoporosis of 50–90% [4,5]. Fractures occur in 30–50% of the cases, being more frequent in men than in women (2), especially in thoracic and lumbar spine [4,5] and are strongly correlated with the degree and duration of hypercortisolism [6]. Dual X-ray absorptiometry (DXA) is the standard non-invasive technique for measuring bone mineral density (BMD) at the lumbar spine (L2–L4) and hip, as it allows the assessment of the risk of fractures if there is osteoporosis, as well as improvement after response to medical treatment or resolution of the cause in secondary osteoporosis.

Vertebral fractures in CD are usually treated conservatively, as every other osteoporotic fracture, and surgical treatment is infrequently needed [7]. Treating the primary cause could prevent new fractures [8].

We present a case with consecutive thoracolumbar vertebral fractures which led to a secondary kyphotic thoracolumbar deformity treated by arthrodesis and corrective posterior osteotomies. This report is line with the SCARE criteria [9].

## 2 Presentation of case

A 43-year-old man was referred to our hospital due to lumbar pain without previous trauma. He was previously diagnosed of refractory arterial hypertension. The initial exam did not reveal neurological deficit. The patient showed signs such as abdominal perimeter increase, abdominal violaceous striae, thin skin, weight loss more significant in the last month, intense proximal weakness in the lower limbs, ecchymosis, toenails, and armpits fungal lesions, erythema and moon facies, all happening over the course of one year. The patient had never taken any corticoids. After that, he was diagnosed of a nosocomial respiratory infection due to influenza A virus treated with oseltamivir.

**Fig. 1:**
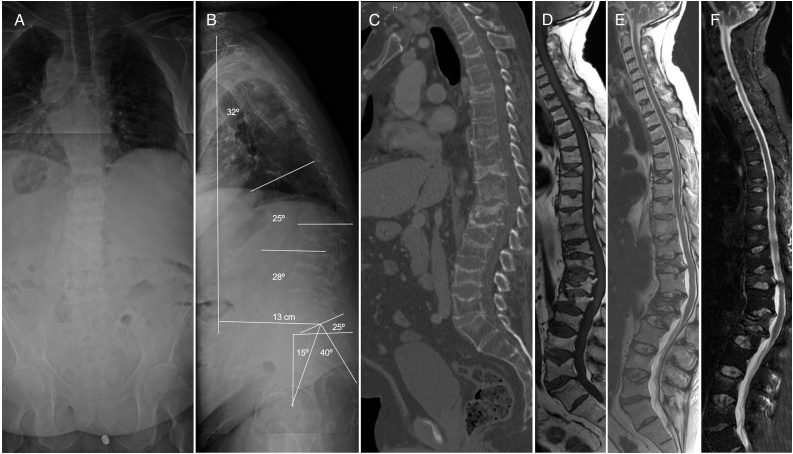
A/B: Standing full spine x-ray preoperative antero-posterior and sagittal view. A: No coronal imbalance or angular deformities showed in AP view. B: Sagittal view: Multiples vertebral fractures were observed in thoracic, lumbar and consecutives in thoracolumbar area leads to TLK (T10-L2) 25° and sagittal vertical axis (SVA) 13 cm. C/D/E/F: TC preoperative sagittal view shows loss of vertebral body height and kyphosis in thoracolumbar area and sagittal views of MR T1, T2-weighted and STIR sequences without oedema suggesting acute lesion.

**Fig. 2:**
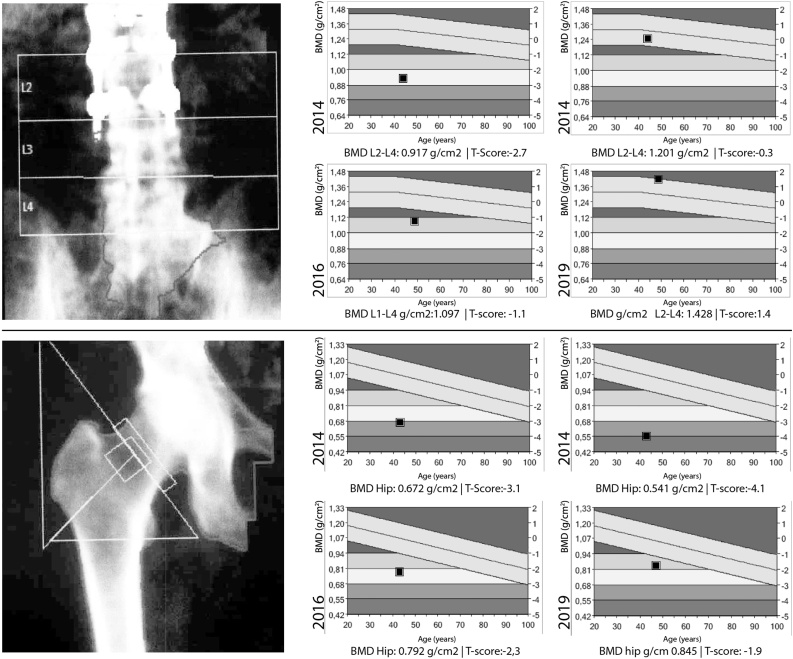
DXA changes through follow-up. Dual energy X-ray absorptiometry (DXA) recovered a normal value in the L2-L4 lumbar spine (T-score 1.4) and an osteopenic value in the hip (T-score -1.9) at six years of follow-up.

X-ray and computerised tomography (TC) showed rib and multiple vertebral compression fractures in thoracic T6 level, thoracolumbar T11, T12, L1, L2 and L4, L5 lumbar levels. Magnetic resonance (MR) confirmed multiple vertebral fractures without malignant signs, which suggested osteoporotic fractures (Fig. 1). Dual energy X-ray absorptiometry (DXA) showed osteoporosis in spine and hip (lumbar spine T-score -2.7 and femoral neck T-score -3.1) (Fig. 2).

When Cushing’s syndrome was suspected, the patient was referred to the endocrinologist for a confirmation study and the following analytic studies were performed. Cortisol rhythm: Serum cortisol 38.4 μg/dl (8 h), 38.7 μg/dl (23 h) (normal value 6–30 μg/dl), Urinary free cortisol: 1616.8 μg/24 h (normal value 4.3–176 μg/24 h), ACTH: 104 pg/mL (normal value 7–63 pg/ml). ACTH-dependent Cushing’s disease was confirmed. Low-dose (0.5 mg every 6 h for two days) and high-dose (8 mg single dose) dexamethasone suppression tests did not suppress cortisol levels (35.8 μg/dl after low dose and 33.7 μg/dl after high dose of dexamethasone). Pituitary function was preserved, except for hypogonadotropic hypogonadism secondary to hypercortisolism. Brain MR identified a 5 mm pituitary microadenoma and bilateral inferior petrosal sinus sampling showed a central to peripheral ACTH gradient (>2 basally and >3 post-ACTH), thus confirming Cushing’s disease.

The patient was initially treated with anti-hypertensive medical therapy, ketoconazole, and testosterone. Moreover, teriparatide treatment was also started to treat osteoporosis. However, the patient started to have spine and neck erythema and non-puriginic painful thorax. He was treated by the dermatologist because of the lesions’ deterioration which had expanded to the trunk, limbs, head, and neck, with some blisters appearing in areas of friction with associated epidermolysis and Nikolsky sign [10]. The biopsy confirmed toxic epidermal necrolysis (TEN) related to medical treatment, probably related to oseltamivir [11].

The patient underwent an endoscopic-assisted endonasal trans-sphenoidal excision of the pituitary adenoma by the neurosurgical team, with hypercortisolism resolution and development of secondary adrenal insufficiency, which required hydrocortisone substitutive treatment for 15 months.

After three months, the patient presented forward inclination of the trunk and had difficulties for standing or walking. CT and MR showed multiple vertebral compression fractures in thoracic T6, thoracolumbar T11, T12, L1, L2 and lumbar L4, L5 levels without cleft or vacuum signs and diffuse hypointense in T1-weighted and T2-weighted sequences, which excluded osteonecrosis. However, X-ray full standing spine showed thoracolumbar kyphosis and disabling sagittal deformity. Spinal parameters were: Sagittal vertical axis (SVA) 13 cm, thoracic kyphosis T2-T12 (TK) 32°, thoracolumbar kyphosis T10-L2 (TLK) 25°, lumbar lordosis L1-S1 (LL) 28°. Pelvic parameters: Pelvic incidence (PI) 40°, sacral slope (SS) 25° and pelvic tilt (PT) 15° (Fig. 1).

Surgical treatment was decided and with general anaesthesia by posterior approach we perform a T10-L3 posterolateral arthrodesis using autologous bone and two-level posterior Smith-Petersen corrective osteotomies (SPO) [12]. Weak intraoperative bone was observed so fenestrated pedicle screws reinforced with PMMA were used to increase the insertional torque and pull-out strength preventing instrumentation failure. The procedure was performed for a main author.

**Fig. 3:**
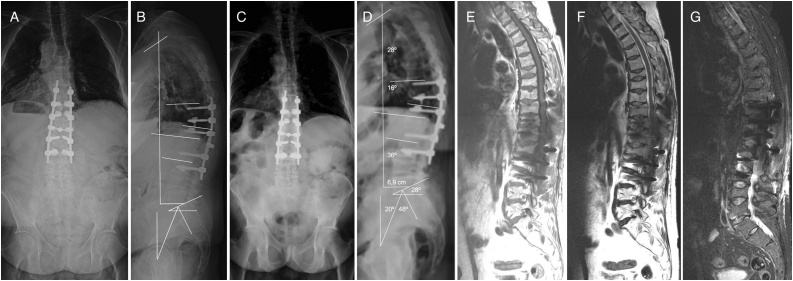
A/B: Standing full spine x-ray postoperative antero-posterior and sagittal view. C/D: Coronal and sagittal views at end of follow-up, showing T10-L3 arthrodesis with fenestrated screws and PMMA, adequate balance, well positioned implants without osteolysis or proximal junctional faillure. E/F/G: MR T1, T2-weighted and STIR sagittal sequences don’t show news fractures at six-years of follow-up.

At six years of follow-up the patient had no standing or walking pain and he recovered his previous working activity. Dual energy X-ray absorptiometry (DXA) recovered a normal value in the L2-L4 lumbar spine (T-score 1.4) and an osteopenic value in the hip (T-score -1.9) at six years of follow-up (Fig. 2). X-ray study showed an adequate SVA 6.9 cm, TK (T2-T12) of 28°, and TLK (T10-L2) 16° and LL of 36° (Fig. 3). Although physiological thoracolumbar kyphosis wasn’t recovered it was well tolerated.

## 3 Discussion

Osteoporosis is a common feature in Cushing’s disease, with a highly variable prevalence (50–90%) and vertebral fractures can be the first clinical manifestation, as it was in our patient [8]. A high number of pathologic vertebral fractures in different locations and evolutive stages, as in our case, have been related to endogenous hypercortisolism bone disease and its vertebral trabecular bone preference [13]. Higher serum cortisol levels and longer duration of hypercortisolism confer a higher fracture risk [6]. Glucocorticoid excess affects osteoblast differentiation and function leading to bone loss and increase in fracture risk. Also, it affects osteocyte number and function, inducing apoptosis and reducing mineral matrix with an increase in lacunar size [14]. As a consequence, there is a disproportionate loss of bone strength in relation to bone mass especially in trabecular sites [13].

Treating the underlying cause reduces the risk of new fractures, but it may persist, since complete bone mineral density recovery is not always achieved [15]. Furthermore, fractures may develop even in the presence of normal or low-normal bone mineral density (BMD). DXA has been proposed as the standard BMD measuring technique. It allows the assessment of initial treatment response as well as long-term recovery. Bone mass recovery after successful treatment of Cushing’s syndrome may take 5–10 years [16,17].

Analgesics and bracing conservative therapy can be useful for isolated or multiple acute osteoporotic vertebral fractures. Reinforcement techniques such as percutaneous vertebroplasty (PVP) or balloon-kyphoplasty (BKP), are generally accepted treatment for vertebral compression fractures, if pain remains after six weeks. Reinforcement techniques, when compared to conservative treatment, achieve better pain relief, functional recovery and quality of life after one-year follow-up, although no differences are found in long-term pain relief [18]. Moreover, thoracolumbar kyphosis may not be corrected or even increase after PVP or BKP in transitional thoracolumbar area [19].

In our case, reinforcement techniques weren’t considered because MR showed multiple vertebral fractures with isointense signal in T1 and T2 weighted sequences suggesting long-term evolution. However, isolated vertebral osteoporotic fractures are more frequent in the thoracolumbar area and can be associated with poor evolution signs with cleft or vacuum signs presence indicating osteonecrosis [20]. These findings weren’t present in our case and malignant signs were ruled out. Furthermore, multiples and consecutive fractures can lead to progressive local or regional deformity causing pain, disability and increase neurologic risk and decompression or realignment techniques associated with instrumentation can be required to restore sagittal balance [21]. Fusion levels and length can be conditioned by the number of fractures in deformity, apex of deformity and the presence of old fractures or previous degenerative changes, in the thoracic or lumbosacral area. Osteotomy type can be chosen depending on deformity shape (whether sharp or round), rigidity and amount of correction [12]. For the treatment of sagittal deformity, the kyphotic thoracolumbar segment was included and in the instrumented upper vertebra election, only one vertebra upper to the fractured one (T11) was elected (T10) to avoid the thoracic apical area and its complications such as proximal junctional kyphosis or proximal junctional failure. The presence of consolidated and without kyphosis compression fracture in T6 allowed us not extending the fusion to the upper thoracic area. When electing the lower instrumented vertebra, we considered L3 as a stable vertebra due to it is below the most compressed inferior vertebra (L2), the need of preserving as many lumbar levels as possible and to avoid extending fusion to S1 due to the presence of degenerative changes in L5-S1 segment.

In this case, apart from thoracic vertebral fractures, multiple consecutive vertebral thoracolumbar fractures led to secondary painful kyphosis of 25° and sagittal imbalance, specially standing or walking. Due to moderate and flexible deformity, Smith-Petersen osteotomies (SPO) were performed at the apex of the deformity with shortening of the posterior column (16° postoperative TLK) and postoperative SVA of 6.9 cm. In the presence of poor bone quality, pedicle screw instrumentation can be unsuccessful due to implant loosening or breaking. In patients with postmenopausal osteoporosis, teriparatide treatment for at least one month prior to surgery can be effective maximizing the purchase of pedicle screws by increasing their insertional torque. However, teriparatide had to be stopped in our patient after a few days of treatment, since he developed TEN. Finally, oseltamivir was confirmed as the cause of epidermal disorder [11]. Reinforcement techniques with PMMA or fenestrated screws decrease pull-out risk and were used in this patient due to his low mineral density [22].

## 4 Conclusion

Vertebral fractures are the most frequent bone lesions associated with Cushing’s disease, which often improve after treatment of this disease. Early diagnosis is important as the high rate of comorbidity and mortality in CD patients can be avoided if treated at an early stage. The conservative therapy can be useful for isolated or multiple osteoporotic vertebral fractures. However, when there is a late diagnosis and/or severe lesion, surgery may be required, in order to address secondary vertebral deformity.

## Declaration of Competing Interest

José Ramírez-Villaescusa has nothing to disclose.

David Ruiz-Picazo has nothing to disclose.

Cristina Lamas-Oliveira has nothing to disclose.

Carlos Morillas-Ariño has nothing to disclose.

## Funding

This Research did not receive and specific grant form funding agencies in the public, commercial, or not-for-profit sectors.

## Ethical approval

The paper is a case report, and therefore does not require ethics approval.

## Consent

Informed consent has been obtained from the patient, and all identifying details have been omitted.

## Author contribution

José Ramírez-Villaescusa: Study concept, acquisition and analysis of data and writing the paper.

David Ruiz-Picazo: Acquisition and analysis of data.

Cristina Lamas-Oliveira: Data analysis interpretation and revision of the article.

Carlos Morillas-Ariño: Data analysis interpretation and revision of the article.

## Registration of research studies

NA.

## Guarantor

José Ramírez-Villaescusa.

## Provenance and peer review

Not commissioned, externally peer-reviewed.
